# Can Children Catch up from the Consequences of Undernourishment? Evidence from Child Linear Growth, Developmental Epigenetics, and Brain and Neurocognitive Development

**DOI:** 10.1093/advances/nmaa020

**Published:** 2020-06-25

**Authors:** Jef L Leroy, Edward A Frongillo, Pragya Dewan, Maureen M Black, Robert A Waterland

**Affiliations:** Poverty, Health, and Nutrition Division, International Food Policy Research Institute, Washington, DC, USA; Department of Health Promotion, Education, and Behavior, University of South Carolina, Columbia, SC, USA; Poverty, Health, and Nutrition Division, International Food Policy Research Institute, Washington, DC, USA; Department of Pediatrics, University of Maryland School of Medicine, Baltimore, MD, USA; RTI International, Research Triangle Park, NC, USA; USDA/Agricultural Research Service Children's Nutrition Research Center, Departments of Pediatrics and Molecular & Human Genetics, Baylor College of Medicine, Houston, TX, USA

**Keywords:** catch-up, linear growth, recovery, undernutrition, undernourishment, adoption, developmental epigenetics, DNA methylation, brain and neurocognitive development

## Abstract

Recovery from nutritionally induced height deficits continues to garner attention. The current literature on catch-up growth, however, has 2 important limitations: wide-ranging definitions of catch-up growth are used, and it remains unclear whether children can recover from the broader consequences of undernutrition. We addressed these shortcomings by reviewing the literature on the criteria for catch-up in linear growth and on the potential to recover from undernutrition early in life in 3 domains: linear growth, developmental epigenetics, and child brain and neurocognitive development. Four criteria must be met to demonstrate catch-up growth in height: after a period in which a growth-inhibiting condition (criterion 1) causes a reduction in linear growth velocity (criterion 2), alleviation of the inhibiting condition (criterion 3) leads to higher-than-normal velocity (criterion 4). Accordingly, studies that are observational, do not use absolute height, or have no alleviation of an inhibiting condition cannot be used to establish catch-up growth. Adoption and foster care, which provide dramatic improvements in children's living conditions not typically attained in nutrition interventions, led to some (but incomplete) recovery in linear growth and brain and neurocognitive development. Maternal nutrition around the time of conception was shown to have long-term (potentially permanent) effects on DNA methylation in the offspring. Undernourishment early in life may thus have profound irreversible effects. Scientific, program, and policy efforts should focus on preventing maternal and child undernutrition rather than on correcting its consequences or attempting to prove they can be corrected.

## Introduction

Child undernutrition continues to be an important global health problem. Not only does undernutrition increase susceptibility to illness and the risk of death, it also contributes to delays in neurocognitive development, low school achievement, reduced earnings in adulthood, and increased probability of adult noncommunicable chronic diseases (1).

Recognizing the debilitating consequences of child undernutrition, the global community has responded by setting targets to reduce the worldwide burden of this problem (2), and reducing the prevalence of child stunting has become a main international nutrition goal (3). Based on current evidence, the most effective strategy to reduce stunting is through programs that prevent (rather than treat or reverse) linear growth retardation during the first 1000 d of life (that is, from conception to the child's second birthday) (1, 4, 5). Notwithstanding the commonly accepted view in the nutrition community that linear growth retardation is largely irreversible outside this 1000-d window, the question of whether a height deficit can be restored continues to garner attention in human biology, nutrition, and development economics.

The current literature on reversal of growth retardation (or “catch-up growth”) has 2 important limitations. First, wide-ranging definitions of catch-up growth are used, creating confusion as to how to identify catch-up growth and as to whether it is possible (6). A second issue relates to the relevance of catch-up growth. Much of the recent literature rests on the premise that linear growth retardation and stunting negatively affect child (neurocognitive) development, and that any recovery from linear growth retardation or stunting will thus automatically lead to improved neurocognitive outcomes. This assumption, however, does not hold: poor linear growth does not cause delays in child development (7, 8). Therefore, evidence on catch-up in linear growth does not provide insights on children's capacity to recover from the consequences of undernutrition on neurocognitive outcomes and possibly other domains relevant to the child's future wellbeing.

Our objective was to address these shortcomings by carefully reviewing the literature on the potential to recover from undernutrition early in life. We focus on 3 domains: linear growth, developmental epigenetics, and child development. We start with stating the definition of catch-up in linear growth and apply this definition to the current literature. We then review whether, in terms of developmental epigenetics and brain and neurocognitive development, recovery from undernutrition is possible.

### Defining catch-up in linear growth

Catch-up growth in individual children was first described in 1963 in children who were treated for secondary growth disorders (such as renal disease and celiac disease). It was characterized as “rapid linear growth that allowed the child to accelerate toward and, in favorable circumstances, resume his/her pre-illness growth curve” (9, 10). More recently, catch-up in linear growth has been defined by Boersma and Wit (10) as “height velocity above the statistical limits of normality for age or maturity during a defined period of time, following a transient period of growth inhibition; the effect of catch-up growth is to take the child towards his/her pre-retardation growth curve.” This definition of catch-up growth implies that 4 criteria must be met to demonstrate catch-up growth in height. First, a growth-inhibiting condition is required (criterion 1) which causes a reduction in linear growth velocity (criterion 2). This period of growth inhibition is followed by alleviation of or compensation for the inhibiting condition (criterion 3) which subsequently leads to higher-than-normal velocity (criterion 4) (6).

The recent literature has used a wide range of definitions of catch-up growth, including a change in height-for-age *z* score (HAZ) >0.67, achieving a HAZ above −2 or −1.6, or reaching height above the third percentile for height (for age) at any time during follow-up (11–21). These various definitions have created substantial confusion in the literature, oversimplify the biology of human growth and development, and create the impression that new descriptions of catch-up growth are simply invented to fit the research methods (6). The primary causes of this problem are a poor understanding of the meaning of “higher-than-normal velocity” and confusion about which velocity to use.

#### Higher-than-normal velocity

**FIGURE 1:**
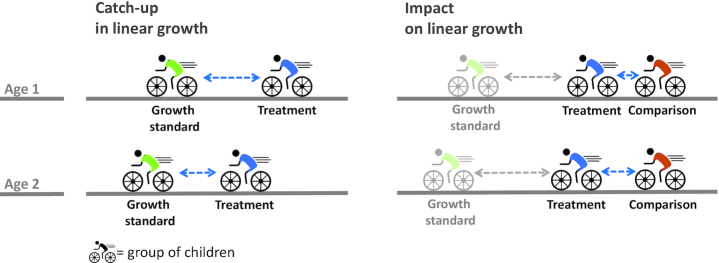
The difference between the requirements for documenting catch-up in linear growth and documenting impact on linear growth. Catch-up in linear growth requires children to grow faster (i.e., at a higher velocity) than expected for their age and sex. A cycling analogy makes this clear: catching up with the green cyclists ahead (in our case, the growth standard) requires the blue cyclists who have fallen behind (in our case, children with growth retardation) to cycle faster than the cyclists ahead do. By covering a larger distance between age 1 and age 2 (in our case, by accumulating more centimeters), the blue cyclists have narrowed the gap with the green cyclists (in our case, the growth deficit has become smaller). Establishing impact of a nutrition intervention on linear growth requires the cyclists in blue (in our case, children receiving the treatment) to cycle faster than the cyclists in orange (in our case, children in the comparison group), thus creating a gap between both groups between age 1 and age 2 (in our case, a difference in height). Because the cyclists in blue (the treated children) may still be cycling slower than the cyclists in green (the growth standard), the gap between both may still have grown over time. Consequently, a positive impact on linear growth of an intervention does not imply that catch-up growth has occurred. Even if the intervention group improved relative to the comparison group, the height deficit of the intervention group relative to the standard may have widened.

The only way to reduce the accumulated height gap is for children to grow faster (i.e., at a higher velocity) than expected for their age and sex (22). Identifying higher-than-normal velocity requires the use of a growth standard which provides information on the expected median and distribution of growth velocity for a given age and sex. Recent literature on catch-up growth ignores this requirement. A cycling analogy (**Figure 1**) illustrates this point: catching up with cyclists ahead requires one to cycle faster than they do. The figure also illustrates that a positive impact on linear growth of an intervention does not imply that catch-up growth occurred. Even if the intervention group improved relative to the comparison group, the height deficit of the intervention group relative to the standard may have widened (6).

#### Velocity of what?

Only absolute height velocity (i.e., the change in height in cm with age) can be used to study catch-up growth. The common use of HAZs [sometimes referred to as “relative catch-up growth” (23)] is incorrect. First, HAZs are constructed using cross-sectional SDs and are inappropriate to study changes in height with age (22, 24); thus, “HAZ-velocities” (i.e., changes in HAZ with age) are not a meaningful construct. Furthermore, the cross-sectional SDs used in the denominator of HAZs increase with age such that a child with a constant absolute height deficit will nevertheless appear to improve with age based on the HAZ. Second, absolute height velocity directly relates to the consequences of linear growth retardation. Contrary to what is commonly believed, only 2 sets of outcomes are caused by linear growth retardation: linear growth retardation causes short stature at adulthood in mothers and this in turns contributes to difficult birth and poor birth outcomes. What matters for these outcomes is the absolute height of the mother and not her relative size (7).

#### Establishing catch-up growth

Documenting catch-up in linear growth thus requires children to gain length or height in absolute terms faster than the expected linear growth velocity for their age and sex. This is mathematically equal to a reduction in the absolute height deficit [or height-for-age difference (HAD); see **Box 1**] with age (22). None of the definitions of catch-up growth recently used in the literature is equivalent to the use of absolute height velocity. The use of these other definitions will thus lead to erroneous conclusions (22).

**BOX 1: HAZ and HAD**

Growth deficits in height in groups of children are expressed as the mean of the individual deficits. These are calculated as the difference between the measured height and the median age- and sex-specific height from the 2006 WHO growth standard (25). This HAD can be used in absolute terms or be used standardized by dividing HAD by the SD from the growth standards to calculate HAZ (24):

$$\begin{eqnarray*}
{\rm{\ HAD}} = {\rm{\ observed\ height}} - {\rm{median\ height\ growth\ standard}}\end{eqnarray*}$$

$$\begin{eqnarray*}
{\rm{\ HAZ}} &=& {\rm{\ \ }}\frac{{{\rm{observed\ height}} - {\rm{median\ height\ growth\ standard}}}}{{{\rm{SD\ growth\ standard}}}}\nonumber\\
&=& \frac{{{\rm{HAD}}}}{{{\rm{SD}}}}{\rm{\ }} \end{eqnarray*}$$

HAZ is constructed using cross-sectional SDs. HAZ is useful to assess the attained height of children at a given age but is inappropriate to assess changes in height as children age; HAZ is thus inappropriate to assess catch-up growth in height (24). Assessing catch-up growth using HAZ is mathematically different from using HAD and has been demonstrated to lead to erroneous conclusions (22).

### Is catch-up in linear growth possible?

Both observational studies and studies assessing the impact of interventions have claimed to assess catch-up growth. We discuss both but limit our review to studies on groups of (rather than individual) children younger than 5 y for 2 reasons. First, a growth standard is available only for children younger than 5 y. Second, the growth standard shows how groups of children are expected to grow. A counterfactual for each individual child, that is, how the child would have grown in the absence of the growth-inhibiting condition, is impossible to establish.

Observational studies, by definition, violate the third criterion to establish catch-up growth, i.e., they do not assess linear growth after the cause of the growth inhibition has been alleviated. Therefore, observational studies cannot be used to establish whether catch-up in linear growth is possible and we do not discuss them further, focusing on experimental or quasi-experimental studies of interventions. We limited our review to adoption studies because they provide the most dramatic improvement in a child's environment with respect to diet, water, sanitation, hygiene, and opportunities for learning and receiving responsive care in a stable household setting. Accordingly, adoption studies provide evidence of the upper bound for what is possible for linear growth outcomes when environmentally inhibiting conditions are alleviated.

We used multiple search strategies to identify articles to include in the review. First, we screened all studies included in the 1994 Martorell et al. (26) seminal review on the reversibility of stunting and screened all studies that have cited this review since it was published using Web of Science. We followed the same backward- and forward-looking strategy using the more recent comprehensive review by van IJzendoorn et al. (27) of studies on plasticity of growth after international adoption published between 1956 and 2006. To identify additional studies examining the link between linear growth and adoption published after 2006, we searched PubMed using the search string “(catch up OR recovery OR growth OR height) AND (adopt OR orphan) AND (child OR infant).” We only included studies that assessed height outcomes (absolute height, HAZ, or height percentiles) in children <5 y of age before and after adoption in order to quantify catch-up growth. A total of 11 studies met the inclusion criteria, providing a total of 13 catch-up growth estimates (**Table 1**, **Supplemental Table 1**). We first computed the children's actual height using the WHO growth standard and the reported sex, mean age, and mean HAZ. We then calculated the difference between the estimated actual height and the expected mean height (the median of the WHO growth standard), i.e., the HAD. We followed this method for the baseline and follow-up values (i.e., before and after adoption). A decrease in HAD from baseline to follow-up provided evidence of catch-up growth, indicating that the children grew faster in their adoptive environment than expected based on the growth standard, thereby reducing the accumulated height gap. Additional details on the methods are provided in the **Supplemental Methods**.

**TABLE 1:** Impact of adoption on height-for-age difference in 11 reviewed studies providing a total of 13 estimates of catch-up growth

		Country of origin	Country of adoption		Mean age, mo			Height-for-age difference, cm		
	Study			*n*	Baseline	Follow-up	Difference	Baseline	Follow-up	Difference
1	Proos et al. (28)	India	Sweden	46	15.2	39.2	24.0	−5.9	−2.7	3.3
2	Melsen et al. (29)	Asia	Denmark	71	21.8	33.8	12.0	−2.7	−3.4	−0.7
3	Oostdijk et al. (30)	Various	Netherlands	94	34.8	46.8	12.0	−6.7	−4.6	2.0
4	Oostdijk et al. (30)	Various	Netherlands	75	34.8	58.8	24.0	−6.7	−3.2	3.4
5	Rutter et al. (31)	Romania	England	58	6.6	48.0	41.4	−4.4	−1.4	3.1
6	Jenista and Chapman (32)	Various	USA	128	6.0	20.0	14.0	−0.8	−1.3	−0.4
7	Esposito et al. (33)	Various	USA	60	26.0	40.5	14.6	−5.0	−2.6	2.4
8	Esposito et al. (33)	Various	USA	46	32.6	49.7	17.1	−2.4	−2.7	−0.2
9	Ferrara et al. (34)	Italy	Italy	33	8.3	32.3	24.0	−2.5	−0.5	1.9
10	Fuglestad et al. (35)	Various	USA	58	12.0	18.0	6.0	−3.6	−2.2	1.3
11	Johnson et al. (36)	Romania	USA	55	21.0	42.0	21.0	−2.5	0.5	3.0
12	Kroupina et al. (37)	Eastern Europe	USA	46	18.9	48.9	30.0	−3.3	0.0	3.3
13	van den Dries et al. (38)	China	Netherlands	92	13.0	19.0	6.0	−1.7	−2.0	−0.2

**FIGURE 2:**
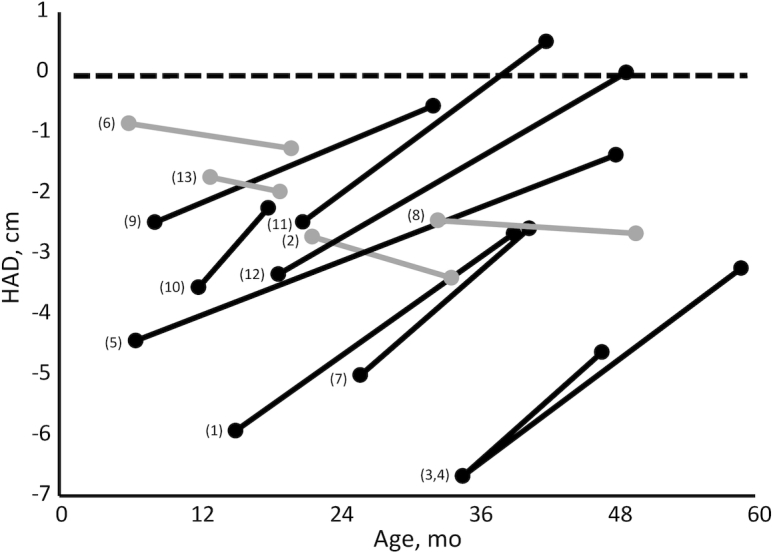
Child HAD before and after adoption in 11 reviewed studies providing a total of 13 estimates of catch-up growth. Black lines correspond to studies in which catch-up in linear growth was found; gray lines show study cohorts in which no catch-up growth was found. Numbers to the left of the baseline value refer to the numbers shown in the left column of Table 1. HAD, height-for-age difference.

Evidence of catch-up in linear growth was found in 9 of the 13 estimates (**Figure 2**, Table 1). The 4 study cohorts with no evidence of catch-up had baseline HAD above−3 cm, suggesting that the potential to catch up might be larger when growth retardation is more severe. Catch-up growth was not limited to children who were adopted before the age of 24 mo, the age after which improvements in linear growth are often considered unlikely (22). Complete catch-up in linear growth was found in only 2 of the study cohorts.

The findings have some inherent limitations that could affect their external validity. The most important limitation is the possibility of selection bias. We do not know which (if any) characteristics or traits made a child more likely to be adopted relative to other children in the same setting. These characteristics (which could be related to their health and nutritional status) may affect their capacity to catch up. We are unable to control for this potential selection in the analyses. Additional limitations are that sample sizes of individual studies were small (with a total of only 855 children across all reviewed studies) and we cannot exclude the possibility that multiple studies included the same adopted children in their analysis. The use of the reported mean values rather than the original individual-level data could have affected the precision because we may have included some observations of children older than 5 y of age (if the reported mean age was <5 y). Finally, we did not have access to the growth reference used in many of the studies and thus used the WHO standard to derive absolute height values from the reported *z* scores.

### Does catch-up in linear growth matter?

The reviewed adoption studies suggest that catch-up growth is biologically possible when children's home environment is dramatically improved. Showing the potential for children to catch up in linear growth contributes to our understanding of the physiology of human growth, but is it relevant for nutrition programs and policy? Nutrition interventions implemented in low- and middle-income countries do not provide the same dramatic improvements in conditions as adoption does. Various nutrition interventions have been shown to improve linear growth (39), but the size of the impact is typically a fraction of that in adoption studies (Table 1) and thus too small for catch-up growth to occur. More importantly, the direct benefits of increasing height early in life are limited to women: taller women have a lower probability of obstructed labor and have better birth outcomes. Other outcomes like child development, work capacity, and noncommunicable disease risk at adulthood are associated with linear growth retardation but are not caused by it (7). Catch-up in linear growth thus should not be expected to lead to gains in these outcomes. Hence, the important question is whether children can recover in other domains after suffering from undernourishment. We reviewed evidence from studies of developmental epigenetics and child development.

### Recovering from the consequences of undernourishment: developmental epigenetics

Long-lasting metabolic consequences of environmental exposures during critical periods of development are broadly referred to as developmental programming (40); this overall paradigm is referred to as the developmental origins of health and disease (DOHaD). Twenty years ago Waterland and Garza (41) proposed the conceptual framework of metabolic imprinting to guide studies into the fundamental mechanisms responsible for the lifelong persistence of these effects. Metabolic imprinting was proposed to encompass adaptive responses to specific nutritional conditions early in life that are characterized by *1*) a susceptibility limited to a critical ontogenic window early in development, *2*) a persistent effect lasting through adulthood, *3*) a specific and measurable outcome (that may differ quantitatively among individuals), and *4*) a dose–response or threshold relation between a specific exposure and outcome. Of 5 proposed potential mechanisms of metabolic imprinting (41), investigators across a range of fields have since made the greatest progress toward understanding metabolic imprinting via nutritional influences on developmental epigenetics.

Epigenetics is the study of mitotically heritable alterations in gene expression potential that are not caused by DNA sequence changes (42). These are the fundamental molecular mechanisms underlying cellular differentiation by which our different somatic cell types, although generally containing the same DNA, stably express very different subsets of genes. Various molecular mechanisms—including DNA methylation, modifications to the histone proteins that comprise the nucleosomes that package DNA in the nucleus, autoregulatory DNA-binding proteins, and noncoding RNA—work together to regulate various locus-specific chromatin states in differentiated cells. Of these, the greatest focus in the DOHaD field has been DNA methylation (43), which is targeted to cytosines within cytosine-guanine dinucleotides (aka “CpG” or “CG” dinucleotides). DNA methylation modulates chromatin conformation and gene expression potential by regulating the affinity of methylation-sensitive DNA-binding proteins. CpG sites are palindromic (i.e., a CpG on the forward strand corresponds to a CpG on the reverse DNA strand). This enables the maintenance of established patterns of CpG methylation during the semiconservative replication of the DNA sequence in which the double-stranded DNA in each daughter cell is formed from 1 existing molecule (which serves as a template) and 1 “new” molecule. Similarly, mitotic heritability of established patterns of CpG methylation is accomplished by the maintenance methylase DNA methyltransferase 1. Hence, cell type-specific patterns of CpG methylation, once established during differentiation, are maintained with high fidelity, leading to the idea that 5-methylcytosine (in the CpG context) may be viewed as the fifth base in the genome. Indeed, CpG methylation is recognized as the most stable epigenetic mark (44), making it a prime candidate to mediate metabolic imprinting.

Studying CpG methylation in humans is challenging for 2 reasons. First, CpG methylation is inherently cell-type specific. Second, it can be difficult or impossible to gain access to the specific cell types thought to be involved in metabolic imprinting, such as those that are involved in the central regulation of energy balance (43). Accordingly, the last 15 y have seen a growing interest in the study of metastable epialleles (MEs) (45), genomic regions at which substantial and systemic interindividual variation in epigenetic regulation occurs stochastically (i.e., rather than being primarily genetically determined). Unlike most of the mammalian genome, at which cell type–specific patterns of CpG methylation are predictably established during cellular differentiation, methylation at MEs is established in a largely stochastic fashion in the very early embryo and then maintained during subsequent differentiation of various cellular lineages. This results in systemic interindividual variation in DNA methylation. The phenomenon of epigenetic metastability was first discovered in mice >50 y ago, when interindividual variation in coat color (46) and tail deformities (47) was observed among inbred (genetically identical) mice. Over the ensuing decades it was understood that these phenomena are due to stochastic interindividual variation in DNA methylation at the *agouti viable yellow* (*A^vy^*) and *axin fused* (*Axin^Fu^*) MEs, respectively (48, 49). When, in these models, Waterland and colleagues demonstrated that maternal promethylation dietary supplementation before and during pregnancy can change offspring coat color (50) and tail kinkiness (51) by increasing DNA methylation at *A^vy^* and *Axin^Fu^*, the potential involvement of MEs in DOHaD became clear.

Subsequent studies have identified candidate MEs in humans (52, 53). Research in subsistence farming communities in the Gambia was instrumental in documenting that, just like murine MEs, establishment of DNA methylation at these loci is influenced by maternal nutrition around the time of conception (54). In these communities, a single annual rainy season results in dramatic seasonal variation in energy expenditure and availability of specific foods. In the context of this natural experiment, Waterland, Prentice, and colleagues focused on a putative ME encompassing the small noncoding RNA *VTRNA2-1* to provide the first human evidence of metabolic imprinting of DNA methylation. Effects on the establishment of methylation at *VTRNA2-1* were shown to occur during a limited period of susceptibility (preimplantation embryonic development), exhibited long-term stability (from childhood to adulthood, and most likely throughout adulthood), and followed dose–response relations with maternal nutritional status biomarkers (riboflavin, methionine, and dimelthylglycine) in early pregnancy (52). Linking patterns of food intake to seasonal variation in maternal nutritional status in these communities is complex, but a recent study documented seasonal variation in maternal intake of riboflavin, folate, choline, and betaine, and wide-ranging seasonal variation in maternal one-carbon nutritional status biomarkers (55). Regarding the specific focus of this article, the limited window of susceptibility to nutritional influence, together with the highly stable nature of CpG methylation, suggests that, at least in the context of MEs, epigenetic recovery from periconceptional malnutrition is not likely. Indeed, providing *A^vy^/a* mice a methyl-supplemented diet for 29 wk postweaning had no effect on coat color or *A^vy^* methylation (56).

Overall, these data on human candidate MEs provide the best evidence that epigenetically mediated metabolic imprinting occurs in humans. Various groups are now exploring these loci, confirming effects of periconceptional exposures on the establishment of methylation, and drawing associations between interindividual variation in DNA methylation and a wide range of phenotypic outcomes related to human disease (57). A recent large-scale unbiased screen for human regions of systemic interindividual epigenetic variation (58), many of which are likely MEs, should enable accelerated progress in understanding how periconceptional nutrition causes metabolic imprinting of DNA methylation and consequent effects on human health and disease.

### Recovering from the consequences of undernourishment: brain and neurocognitive development

Advances in children's neurocognitive development occur through maturation and gene–environment interactions beginning at or before conception (59). These species-specific experiences are programmed to occur during critical and sensitive age periods, referring to periods of heightened sensitivity to specific stimuli. In the case of a critical period, the timing window is relatively inflexible, meaning that if the stimulus does not occur before the window of sensitivity closes, irreversible damage occurs (60). For example, a child who is unable to hear during the first year of life owing to a severe hearing impairment is at significant risk of suffering irreversible damage and permanent hearing loss even if the impairment is subsequently repaired, because sensory input is required for proper development of the auditory cortex (61). In contrast, sensitive time periods are less rigid and the period of sensitivity to specific stimuli is less well defined.

Nutrition plays important roles throughout children's neurocognitive development, often aligned with critical and sensitive time periods (62). The closing of the neural tube, which begins at ∼17–18 d after conception, before most women know that they are pregnant, is often cited as an early example of the interplay between nutrition and development. Folic acid deficiency has been shown to be associated with neural tube disorders, increasing the risk of irreversible conditions including spina bifida and anencephaly. Iodine deficiency during pregnancy and infancy can impair brain development and neurocognition irreversibly and increase infant mortality (63). Nutritional deficiencies often occur in the context of poverty and associated stresses, making it difficult to isolate the effects of nutritional deprivation on neurocognitive development (64). Poverty has long been associated with disadvantages in children's neurocognitive development and in school performance, often thought to be attributed to lack of environmental resources. Recent evidence has shown associations between poverty and reduced volumes of gray matter (principally neuronal cell bodies, associated with processing and cognition, and glia), particularly in areas of the brain associated with learning, including the frontal and temporal cortex and the hippocampus (65).

Poverty is often associated with increased stressors, including chaos, violence, noise, and lack of consistent structure. These stressors have been associated with disruptions to the neuroendocrine system, leading to dysregulation of the stress response system and to deficits in neurocognitive, emotional, and behavioral functioning (66). Thus, poverty may disrupt neural processing and undermine the development of executive function and the regulation of emotion and attention (67).

Nurturant caregiving has been found to be effective in promoting children's neurocognitive development and alleviating some of the negative consequences of early poverty. In a study among preschoolers, caregiving nurturance mediated the associations between poverty and the development of the hippocampus, suggesting that caregiving can have a protective role, even at a neural level (68).

The Bucharest Early Intervention Project provides an extreme example of the impact of severe deprivation on early brain development. Children who had been placed in orphanages at birth were enrolled into a randomized controlled trial between the ages of 6 and 31 mo and randomly assigned into a foster care placement or retained in the institution. A third group of children who had not been institutionalized was recruited as a community comparison. Children placed in foster care experienced complete catch-up in linear growth by 42 mo of age (36) (Table 1, Figure 2). Neural function was assessed with electroencephalograms at enrollment (mean age 22 mo) and followed over time. At age 8 y, children who had been institutionalized had significantly less gray matter than children in the community comparison group, with no differences between children who remained in the institution or were placed in foster care (69). When white matter (principally myelinated axons, connections associated with learning) was measured, children who had been institutionalized had significantly lower volumes than children in the community comparison group. The children who remained in the institution had the lowest mean volumes (significantly lower than the community comparison group); intermediate volumes were found in the foster care group (not significantly different from the community comparison group).

The findings suggest differential effects of environmental deprivation and stress on gray and white matter. Under typical conditions, gray matter volume decreases with age and white matter volume increases. Gray matter volume did not develop as expected during institutionalization and was not responsive to foster care placement. In contrast, although there appears to be a delay in the development of white matter volume associated with early institutionalization, there was some (but not complete) recovery among the foster care group.

When the cognition of children in the Bucharest Early Intervention Project was assessed, children randomly assigned to the foster care group showed improvements at 48 and 54 mo (70). Their scores were significantly lower than those of the comparison group children, however, illustrating incomplete recovery. Furthermore, adults adopted to the United Kingdom as children from orphanages in Romania had abnormal brain structure that explained in part both lower intelligence quotient and greater symptoms of attention-deficit and hyperactivity disorder (71). Taken together, these findings suggest that *1*) early environmental stress associated with institutionalization affects brain development, structure, and function; *2*) complete catch-up in linear growth does not necessarily reflect recovery in other domains; *3*) the possibility of recovery varies, potentially depending on the specific brain structure and function, timing, and the environmental intervention; and *4*) complete recovery in complex functions such as neurocognitive development is difficult even with a comprehensive intervention such as foster care. Because neurocognitive development advances through a predictable sequence of interdependent skills, disparities are likely to increase with age as more sophisticated skills are needed, as shown by the threats to academic success that occur among children without basic literacy skills. Although recovery may be possible in some cases, investment in preventing neurocognitive decline is likely to yield stronger and more long-lasting benefits.

## Discussion

Undernourishment early in life has profound irreversible effects across the 3 domains we reviewed (linear growth, developmental epigenetics, and child development). First, adoption, which provides a dramatic improvement in the living conditions of children, can lead to catch-up in linear growth, but that catch-up growth was not complete in most cases. Adopted children were still shorter than children growing up in ideal circumstances. Second, studies conducted in subsistence communities in the Gambia provide strong evidence of epigenetically mediated metabolic imprinting in humans. Maternal nutrition around the time of conception was shown to have permanent effects on DNA methylation in the offspring. Third, the field of child development has long recognized the permanent damage that occurs if a stimulus or other input (such as nutrition) is not received within a critical time period. Folic acid deficiency around the time of conception causes spina bifida and anencephaly, 2 irreversible conditions. The Bucharest Early Intervention Project showed lasting effects of institutionalization on children's gray and white matter volumes and on neurocognitive performance. Foster care led to complete catch-up in linear growth but could only partly reverse the brain and neurocognitive effects.

What are the implications for policies and programs? First, the reviewed evidence confirms the paramount importance of ensuring adequate nutrition, health, and responsive care from before conception and throughout childhood. If these conditions are not met, irreversible damage is likely to occur (72). Exposure to micronutrient supplementation or balanced energy–protein supplements during pregnancy, which typically does not start before the third month of gestation, comes too late to prevent effects on the offspring's DNA methylation. Recovery in linear growth and development has been documented through adoption and foster care but is only partial. Second, the Bucharest Early Intervention Project shows that even if (partial) recovery is possible in 1 domain, it should not be expected to be possible in other domains. Drastically improving conditions postnatally has the potential to partly alleviate the delays in linear growth and neurocognitive development; it will not, however, change the permanent marks of periconceptional undernutrition on the offspring's DNA methylation. Third, the decreasing plasticity with age lies at the basis of targeting nutrition, health, responsive caregiving, early learning, and security and safety interventions to the first 1000 d. Despite the evidence described here, the inability to recover from (nutrition) insults incurred in these critical windows early in life should not lead to a sense of fatalism, i.e., the feeling that interventions beyond this age will not contribute to the child's nutritional status, health, development, and wellbeing. The evidence from the adoption studies reviewed here suggests that linear growth can respond to interventions after 24 mo of age. Child development is characterized by a successive series of sensitive and critical periods. Each of them provides the opportunity to intervene and have a positive effect. The human brain continues to develop throughout childhood, adolescence, and early adulthood, and evidence has shown that some cognitive skills (abstract reasoning) are not acquired until adolescence (73).

Several important research questions remain. First, documenting the phenotypic impact of epigenetic variation caused by periconceptional exposure to inadequate nutrition will provide invaluable insights into how diseases later in life can be prevented through preconceptional interventions. Second, we need to better understand to what extent specific interventions can compensate for the irreversible damage already incurred. A relatively straightforward example relates to obstructed labor (and its sequelae). This condition, partly caused by linear growth retardation during the mother's own development, can be prevented nearly entirely by means of cesarean delivery or other instrumental delivery (D Walker, UCSF, personal communication 2019). Although challenging, increasing access to and timely use of high-quality obstetric care in developing countries is possible. Other examples come from home- and classroom-based interventions which have been effective in helping children develop compensatory mechanisms to overcome early adversities. Postnatal maternal support has been shown to be effective in altering the intergenerational transmission of stress among infants born to mothers with a history of adverse childhood experiences. Specifically, the transmission of maternal hypothalamic–pituitary–adrenal axis function to infants was moderated by maternal support, as indicated by infant cortisol reactivity (74). Classroom interventions have shown that embedding self-regulation into academic lessons among kindergarten children in high-poverty communities can enhance children's engagement in learning, with beneficial effects on executive functions, reasoning, attention, and salivary cortisol, as well as on academic performance (75). Benefits lasted through early elementary school, suggesting that children acquired basic mechanisms to promote learning.

## Conclusion

The evidence from the 3 domains we reviewed shows that adverse conditions including undernutrition early in life have irreversible effects. Although partial catch-up is possible in linear growth and neurocognition after adoption, most interventions in low- and middle-income countries do not achieve these dramatic improvements in conditions. We maintain that studies that purport to study catch-up using observational data or methods that do not cover the 4 criteria outlined here are inaccurate at best and likely counterproductive. Scientific, program, and policy efforts in nutrition should focus on preventing maternal and child undernutrition rather than on correcting its consequences or attempting to prove that the consequences can be corrected.

## Supplementary Material

nmaa020_Supplemental_File

## Notes

Supported by the CGIAR Research Program on Agriculture for Nutrition and Health (A4NH), led by the International Food Policy Research Institute (to JLL); National Institute of Diabetes and Digestive and Kidney Diseases grant R01 DK106424 (to MMB); and USDA/Agricultural Research Service grant CRIS#3092-5-001-059 (to RAW).

Author disclosures: The authors report no conflicts of interest.

The funders played no role in the design, implementation, analysis, and interpretation of the data.

Supplemental Table 1 and Supplemental Methods are available from the “Supplementary data” link in the online posting of the article and from the same link in the online table of contents at https://academic.oup.com/advances/.

Abbreviations used: *Avy*, agouti viable yellow; *AxinFu*, axin fused; DOHaD, developmental origins of health and disease; HAD, height-for-age difference; HAZ, height-for-age *z* score; ME, metastable epiallele.
