# Supplementary material for: Can Children Catch up from the Consequences of Undernourishment? Evidence from Child Linear Growth, Developmental Epigenetics, and Brain and Neurocognitive Development
Source: Adv Nutr. 2020 Jun 25;11(4):1032–41. doi: 10.1093/advances/nmaa020 (PMC7360439; doi:10.1093/advances/nmaa020)
Supplement: nmaa020_Supplemental_File [file nmaa020_supplemental_file.docx]

**Can children catch up from the consequences of undernourishment? Evidence from child linear growth, developmental epigenetics, and brain and neurocognitive development**

**Jef Leroy et al.**

**Online supplementary material**

**Supplementary Table 1:** Adoption studies identified by the search strategy

| **Search strategy** | **Studies identified** | **Shortlisted based on title and abstract** | **Included** |
| --- | --- | --- | --- |
| Martorell et al. (24) reference list | 37 | 7 | 1 |
| Citations of Martorell et al. (24) | 67 | 4 | 0 |
| van Ijzendoorn et al. (25) reference list | 33 | 10 | 4 |
| Citations of van Ijzendoorn et al. (25) | 94 | 30 | 6 |
| Keyword search on PubMed | 269 | 0 | 0 |
| **Total** |  |  | **11** |

**Supplementary text**

**Is catch-up in linear growth possible?**

**Additional text on methods**

One study examined children placed into foster care. Foster care, while different from adoption, presents a similar change in environment and alleviation of inhibiting conditions for children’s growth (57). Two of the 11 studies identified during the literature review contributed two separate data points to our analyses. The Oostdijk et al. study provided data on growth in children’s height for two time periods (67). We treated them as separate data points to estimate catch-up growth in the same children but over different age intervals. Esposito et al. studied growth in children adopted from overseas post institutionalized settings and in children adopted from international foster care settings (70). These estimates were considered as separate data points as well, resulting in an n of 13.

The change in HAD for each of the 13 data points was calculated separately for boys and girls and then averaged.
